# High throughput sequencing of RNA transcriptomes in *Ruditapes philippinarum* identifies genes involved in osmotic stress response

**DOI:** 10.1038/s41598-017-05397-8

**Published:** 2017-07-10

**Authors:** Hongtao Nie, Liwen Jiang, Peng Chen, Zhongming Huo, Feng Yang, Xiwu Yan

**Affiliations:** 1 College of Fisheries and Life Science, Dalian Ocean University, Dalian, 116023 China; 2 Engineering Research Center of Shellfish Culture and Breeding in Liaoning Province, Dalian Ocean University, Dalian, 116023 China

## Abstract

*Ruditapes philippinarum*, is an economically important marine bivalve species. The ability to cope with low salinity stress is quite important for the survival of aquatic species under natural conditions. In this study, the transcriptional response of the Manila clam to low salinity stress was characterized using RNA sequencing. The transcriptomes of a low salinity-treatment group (FRp1, FRp2), which survived under low salinity stress, and control group (SRp1, SRp2), which was not subjected to low salinity stress, were sequenced with the Illumina HiSeq platform. A total of 196,578 unigenes were generated. GO and KEGG analyses revealed that signal transduction, immune response, cellular component organization or biogenesis, and energy production processes were the most highly enriched pathways among the genes that were differentially expressed under low salinity stress. All these pathways could be assigned to the following biological functions in the low salinity tolerant Manila clam: signal response to low salinity stress, antioxidant response, intracellular free amino acid transport and metabolism, energy production and conversion, cell signaling pathways, and regulation of ionic content and cell volume. In summary, this is the first study using high-throughput sequencing to identify gene targets that could explain osmotic regulation mechanisms under salinity stress in *R. philippinarum*.

## Introduction

Salinity is one of the most important environmental factors. Being relatively constant in open seas, it varies considerably in intertidal zones, estuaries and other biotopes. Salinity has been long recognized as one of the fundamental factors affecting marine species distribution and influencing physiological processes of marine and estuarine organisms, such as survival, hemolymph osmolarity, and tissue water content^1–3^. Organisms may be subjected to hyperosmotic or hypoosmotic stress due to heavy rains or water evaporation during low tide. Thus, the tolerance limit of mollusks is important in coping with salinity change. In recent years, due to global warming, the huge amounts of freshwater inflow disproportionately affect the seas and oceans. Salinity of the superficial water and inshore water decreased acutely in rainy season, which can incur the increasing mortality outbreaks and distribution shifts of marine species. Therefore, salinity is a limiting factor to the survival and distribution of many marine organisms, especially as it varies downward^4^. Most marine invertebrates, as osmoconformers, have blood osmolarities close to that of seawater, lacking the ability to regulate the osmotic pressure of the internal medium^5^.

Many commercially important aquatic animals are sensitive to low salinity stress, and mass mortality is often caused by heavily rainfall in summer^2, 3^. Therefore, it is very important for both scientific researchers and fisheries to investigate the mechanisms underlying the low salinity tolerance of aquaculture species. An increasing number of studies have characterized the acclimation responses of aquaculture animals to low salinity stress, and it has been shown that aquatic animals can gradually shape their salinity adaptive phenotypes with extensive biochemical, metabolic, and physiological acclimation processes, and the signaling transduction, ion transport and transcription regulation are likely involved in the adaptive process to hypo-osmotic conditions^5, 6^.

Researchers have characterized the transcriptional responses elicited by low salinity stress in a number of aquatic animals, including the *Crassostrea gigas* ^7, 8^, *C. hongkongensis* ^6^, *Penaeus monodon* ^9^, *Litopenaeus vannamei* ^10^, *Portunus trituberculatus* ^11^, and *Eriocheir sinensis* ^12^. These studies have identified a large number of osmoregulation-related genes involved in a variety of biological processes that are associated with acclimation to both acute and chronic salinity variations. In addition, some important salinity stress-related genes have also been identified in comparative transcriptomic analyses of *C. gigas* and *C. hongkongensis* ^6^. However, there have been few transcriptomic studies of marine mollusk species to identify and characterize their salinity stress induced gene expression. The tolerance of mollusk is determined by cellular mechanisms of adaptation. Reversible changes of protein and RNA synthesis, alteration of the pattern of multiple molecular forms of different enzymes, and the regulation of ionic content and cell volume were shown to be of importance for the above mentioned mechanisms. The efficiency of resistance and tolerance adaptations to salinity changes may vary in different species and in different colour phenotypes of the same species^13^.

The Manila clam, *Ruditapes philippinarum*, is an economically important marine bivalve species of the aquaculture industry, which has a wide geographic distribution from Europe to Asia^14, 15^. It is reported that Manila clam showed a great capacity to adapt to the new environment^16^. The ability to cope with abiotic and biotic stresses is vital to survival of the clams because of their intertidal benthic inhabiting lifestyle. The Manila clam displays different shell color strains in its natural habitat, which have been selected and cultivated for several generations through clam selective breeding programs^14^. Evidences indicated that zebra and orange strains had stronger resistance to environmental stressors, such as temperature and salinity^14^. It has long been an interesting question how the manila clam could be survived in response to the environmental stresses involved in the fluctuation of salinity. Many studies have been performed to reveal the alterations of mRNA expression^17–20^ and protein expression^21–24^ under abiotic and biotic stresses in the oyster^25^, while expression profiling of miRNAs under osmotic stress remains largely unexplored in clams.

In this study, a high throughput transcriptome sequencing (RNA-seq) approach was adopted to investigate the transcriptome profiles of the gills from *R. philippinarum* adapted to optimal and low salinity seawater. The study aimed to investigate the expression patterns of the different treatment to better understand the transcriptomic regulation in *R. philippinarum* to low salinity stress and identify genes involved in osmoregulation of the Manila clam. This study provides an important resource for future investigations on mechanism of tolerance to hypo-osmotic stress for marine invertebrates.

## Results

### Sequencing and assembly

**Figure 1:**
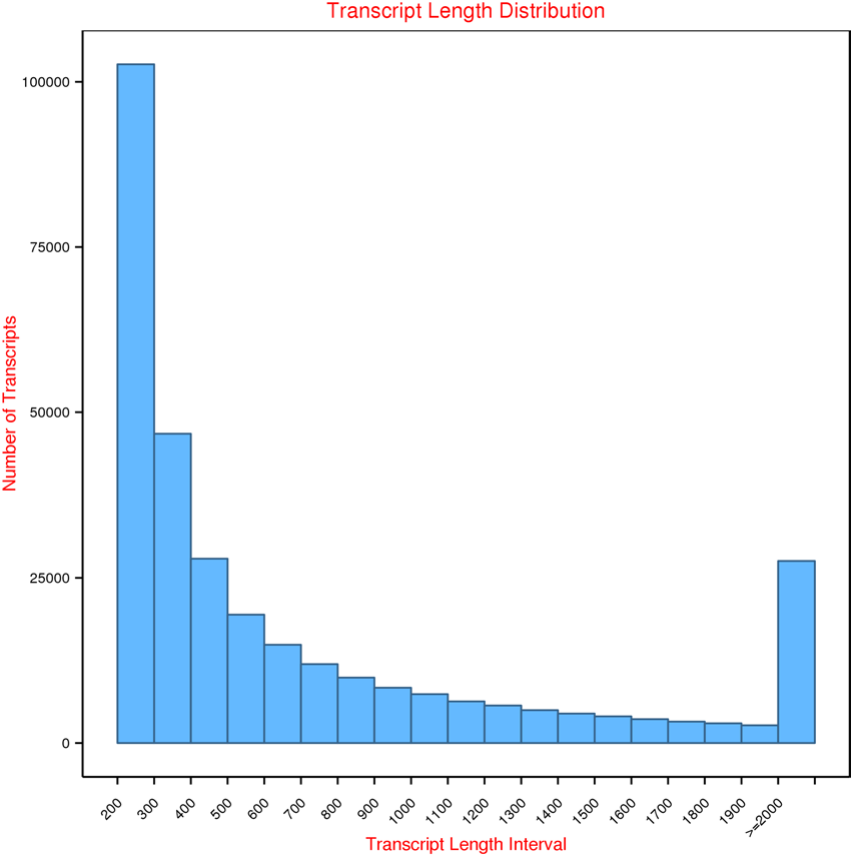
Sequence length distribution of transcripts and unigenes assembled from Illumina reads of the gill transcriptome of *Ruditapes philippinarum*. The x-axis indicates the lengths of the transcripts and unigenes, and the y-axis indicates the number of transcripts and unigenes in each size category.

The transcript length distribution and the number of transcripts are given in Fig. 1. A total of 249,482,420 raw reads with an average length of 125 bp were acquired in the four libraries FRp1, SRp1, FRp2 and SRp2. After the low-quality reads were filtered from the sequence data, 245,015,856 (98.2%) high-quality reads remained and were assembled de novo. The raw reads produced for FRp1, SRp1, FRp2 and SRp2 have been submitted to the NCBI SRA database under accession numbers SRP082480. Because no reference genome exists for *R. philippinarum*, the high-quality reads from the four libraries were combined and assembled into a reference transcriptome with the Trinity software^26^. This assembly yielded 196,578 unigenes with an average length of 664 bp, a minimum length of 201 bp, and a maximum length of 45,933 bp, with an N50 length of 1117 bp (Table 1). A total of 303 groups of eukaryotic BUSCO genes were searched against *R. philippinarum* assembly using the mode of trans with default settings. 293 of the 303 BUSCO genes were found to be present in *R. philippinarum*, suggesting this assembly covers most of the genes of the clam. Summarized benchmarks in BUSCO notation: C: 96% [D: 22%], F: 2.3%, M: 0.9%, n: 303. C: the percentage of complete genes [D: the percentage of duplicated genes], F: the percentage of fragmented genes, M: the percentage of missing genes, n: number of genes used.

**Table 1:** Summary statistics of *Ruditapes philippinarum* transcriptome assembly using Trinity software.

Unigene	Min. Length (bp)	Mean Length (bp)	Max. Length (bp)	Median Length (bp)	N50	N90	Total Length (bp)
Number	201	664	45,933	340	1117	258	130,604,832

### Annotation of Unigenes

**Figure 2:**
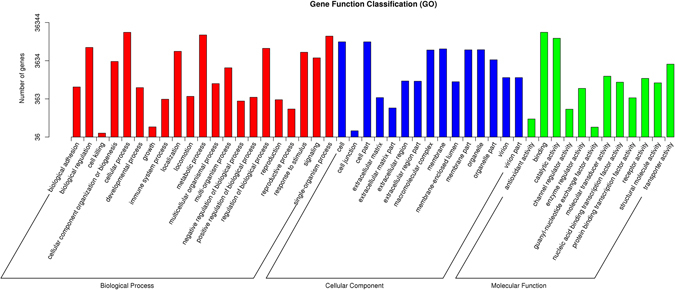
GO (Gene Ontology) categorization (biological process, cellular component, and molecular function) of the unigenes in the gill transcriptome of *R. philippinarum*. Each annotated sequence is assigned at least one GO term.

**Figure 3:**
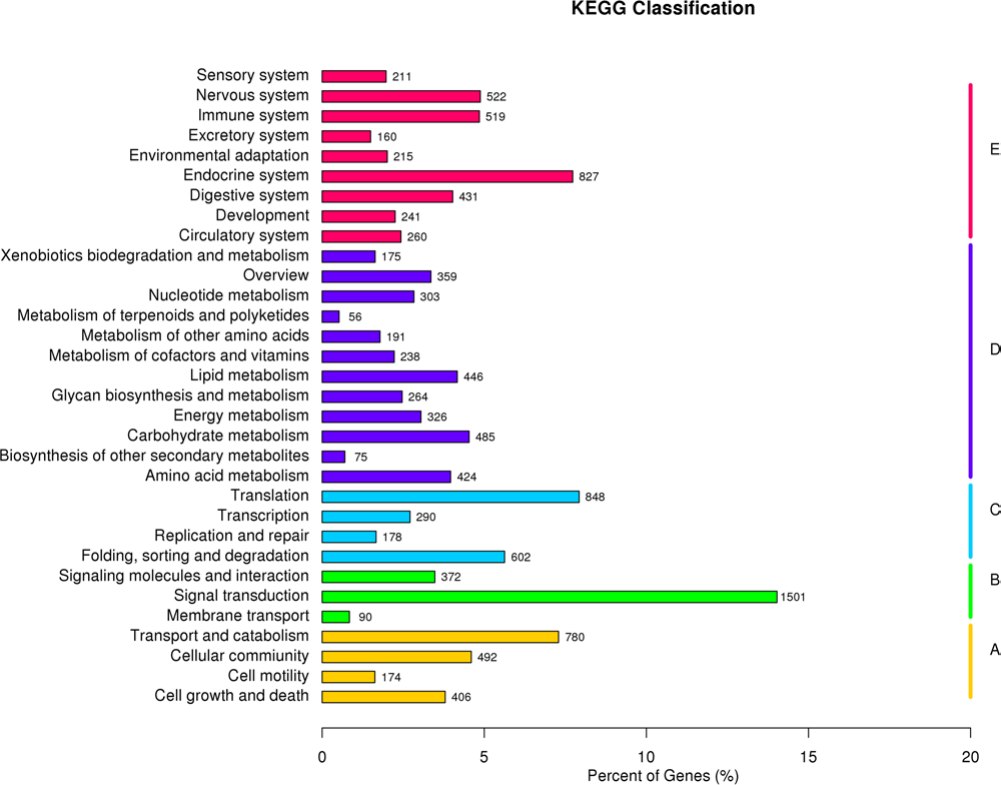
KEGG (Kyoto Encyclopedia of Genes and Genomes) assignment of unigenes in the gill transcriptome of *R. philippinarum*. (**A**), Cellular processes; (**B**) Environmental information processing; (**C**) Genetic information processing; (**D**) Metabolism; (**E**) Organismal systems.

**Figure 4:**
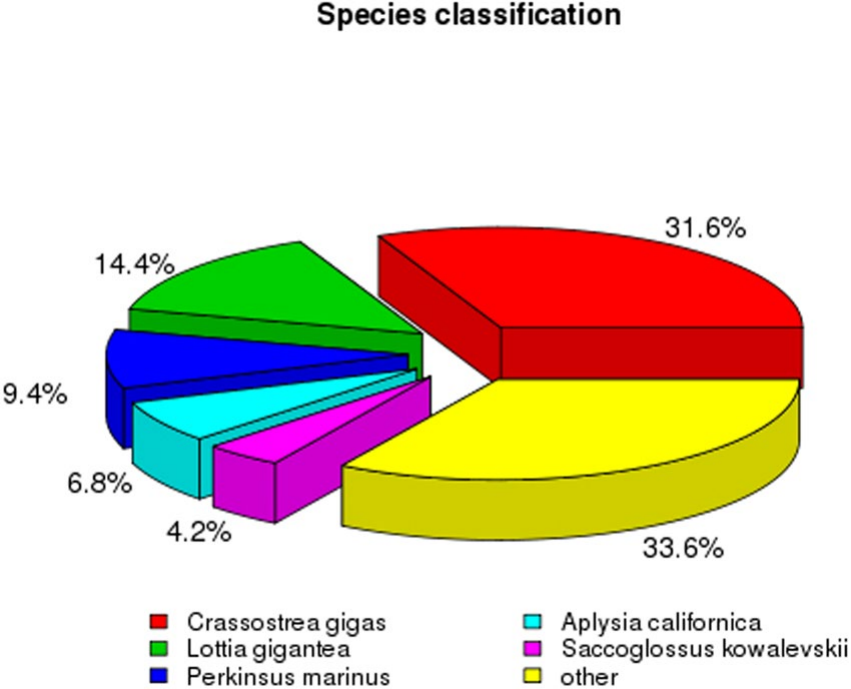
Species distribution of sequences matched to the Nr database.

A total of 196,578 unigenes were annotated by matching them against the Nr and Swiss-Prot databases using a BLASTX search with an E value of 1.0e^−5^; 33,588 unigenes (17.08% of the total) were matched to the Nr database, and 22,122 (11.25% of the total) were matched to the Swiss-Prot database. All the annotated unigenes were used to determine the GO terms and KEGG pathways (Table 2 and Figs 2, 3). In the species distribution of sequences matched to the Nr database, 31.6% of the matched unigenes showed similarities to sequences of *Crassostrea gigas*, followed by *Lottia gigantea* (14.4%), *Perkinsus marinus* (9.4%), *Aplysia californica* (6.8%), *Saccoglossus kowalevskii* (4.2%), and others (33.6%) (Fig. 4).

**Table 2:** Summary statistics of functional annotation of *R. philippinarum* transcriptome.

Category	All sequences	300–999 bp	>=1000 bp
Total number of unigene	196578	83356	31158
Unigene matches against Nr and Swiss-Prot	22053	5579	13962
Unigene matches against GO	36344	12148	17228
Unigene matches against KEGG	10698	2782	6560

### Differentially Expressed Genes

**Figure 5:**
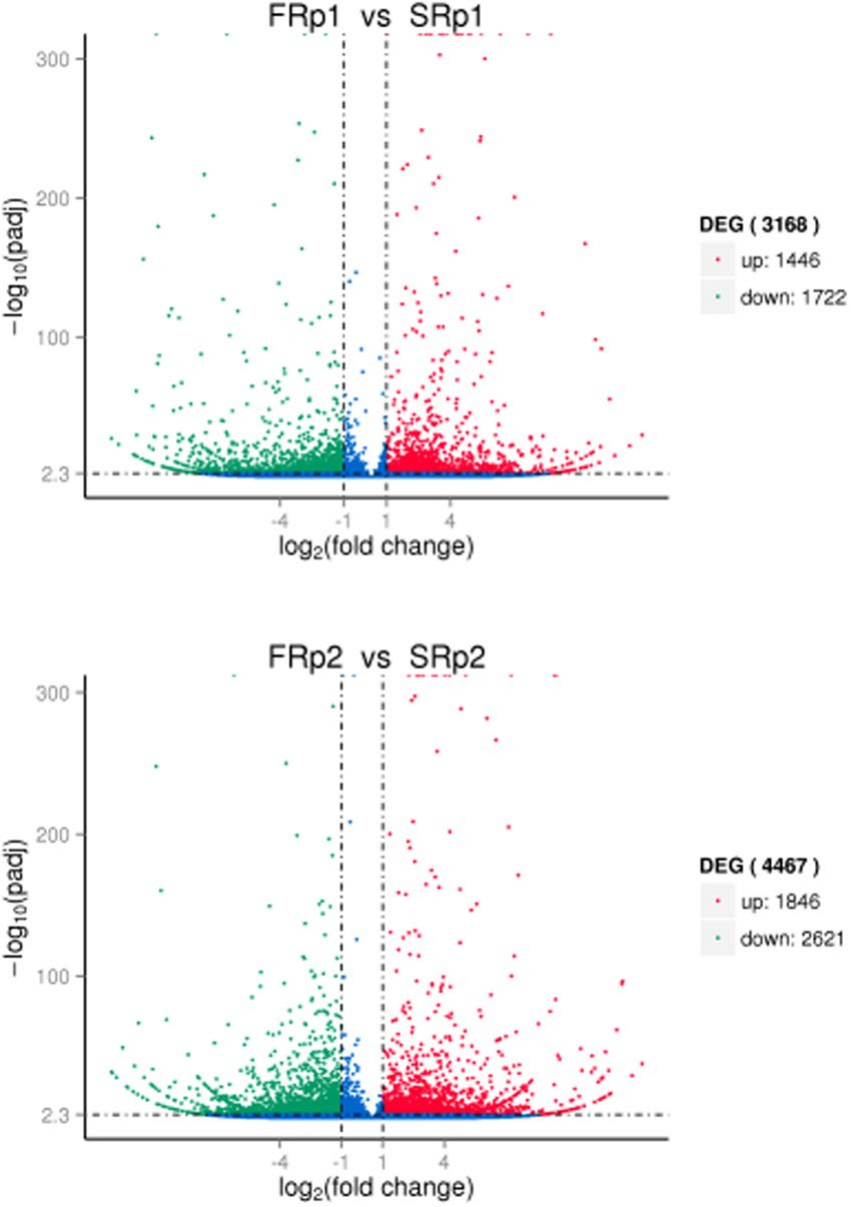
Volcano plot of differentially expressed genes (DEGs) from the transcriptomes of FRp1 vs SRp1 and FRp2 vs SRp2 in *R. philippinarum*.

We obtained 115,733,418 and 129,282,438 qualified Illumina read pairs from the low salinity challenged (FRp1, FRp2) and unchallenged control (SRp1, SRp2) *R. philippinarum* cDNA libraries, respectively. A comparison of this gene expression showed that 4,467 and 3,168 unigenes were differentially expressed after the low-salinity challenge (qvalue < 0.005 and |log2(foldchange)|>1), including 1,846 and 1,446 up-regulated, and 2,621 and 1,722 down-regulated unigenes differentially expressed between FRp1 and SRp1, and FRp2 and SRp2, respectively (Fig. 5). To validate our Illumina sequencing results, 12 differentially regulated genes were selected for RT–qPCR analysis, in which 11 of the genes agreed well with the Illumina sequencing results. The other gene, encoding Rho guanine nucleotide exchange factor 12 (ARHGEF12), did not match the Illumina sequencing results. Therefore, the majority of low salinity responsive genes identified with the Illumina sequencing analysis were consistent with the results of quantitative real-time PCR experiments.

### Gene Ontology (GO) classification

We conducted a GO analysis of the Illumina sequences based on their similarities to known proteins in the Nr database. This analysis provided hierarchical relationships that provide information on the molecular functions, cellular components, and biological processes involved in the low salinity adaptation of the Manila clam (Table 3). A total of 36,344 unigenes were annotated with the GO analysis, with one or more GO term. Of these, 22,287 unigenes were annotated with molecular functions, 23,039 with biological processes, and 14,647 with cellular components. For the cellular components, the major categories represented were cell (GO: 0005623) and cell part (GO: 0044464). For the biological processes, cellular process (GO: 0009987) was the most strongly represented GO term, followed by metabolic process (GO: 0008152). Genes involved in other important biological processes, such as regulation of biological process, localization, single-organism process, response to stimulus, and biological regulation were also identified. A number of unigenes were also involved in interesting categories, such as signaling, membrane, transporter activity, receptor activity, macromolecular complex, cellular component organization or biogenesis, and molecular transducer activity, which may play roles in the low-salinity resistance and adaptation of *R. philippinarum*. Genes involved in binding (GO: 0005488) and catalytic activity (GO: 0003824) were highly represented among the molecular functions.

**Table 3:** GO classification of the differentially expressed genes from *R. philippinarum*.

Term	Comparison	Type	Gene	p-Value
aminoglycan metabolic process (GO:0006022)	FRp1 vs SRp1	Biological process	37	4.86E-05
chitin binding (GO:0008061)	FRp1 vs SRp1	Molecular function	22	4.86E-05
extracellular region (GO:0005576)	FRp1 vs SRp1	Cellular component	220	4.86E-05
chitin metabolic process (GO:0006030)	FRp1 vs SRp1	Biological process	22	4.86E-05
glucosamine-containing compound metabolic process (GO:1901071)	FRp1 vs SRp1	Biological process	22	5.35E-05
amino sugar metabolic process (GO:0006040)	FRp1 vs SRp1	Biological process	25	7.53E-05
carbohydrate derivative binding (GO:0097367)	FRp1 vs SRp1	Molecular function	24	0.000239
calcium ion binding (GO:0005509)	FRp1 vs SRp1	Molecular function	110	0.00306
carbohydrate binding (GO:0030246)	FRp2 vs SRp2	Molecular function	62	0.002591
calcium ion binding (GO:0005509)	FRp2 vs SRp2	Molecular function	154	0.020968

### EuKaryotic Orthologous Groups (KOG) classification

**Figure 6:**
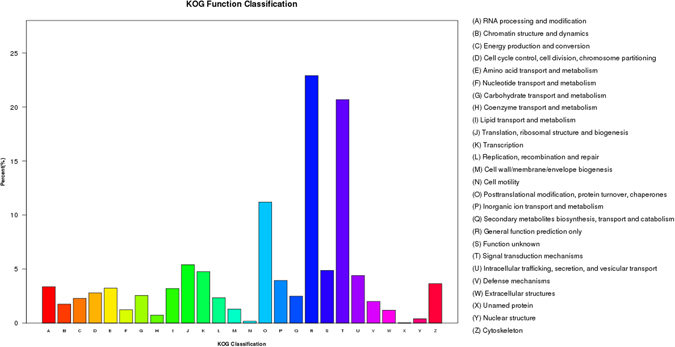
KOG (euKaryotic Ortholog Groups) classifications of putative proteins in the gill transcriptome of *R. philippinarum*.

A KOG analysis was performed to provide a deeper understanding of the functions of the unigenes. About 15,716 unigenes were classified into 26 functional categories. The category ‘General function prediction only’ contained the largest number of unigenes (3,599, 22.90%) (Fig. 6), followed by the ‘signal transduction mechanisms’ cluster (3,250, 20.68%) and the ‘posttranslational modification protein’ cluster (1760, 11.20%). The categories of greatest interest in this study were amino acid transport and metabolism (508, 3.23%), inorganic ion transport and metabolism (620, 3.95%), and energy production and conversion (359, 2.28%). Because the genes in these categories are probably related to intracellular free amino acid transport and metabolism, energy production and conversion, cell signaling pathways, and regulation of ionic content and cell volume, these categories should be considered when molecular markers are developed for marker assisted selection for low salinity tolerant genes in *R. philippinarum*.

### Kyoto Encyclopedia of Genes and Genomes (KEGG) classification

A KEGG pathway analysis was performed of the assembled unigenes to identify the biochemical pathways operating in *R. philippinarum*. The results classified 10,698 unigenes into 270 different pathways, including cellular processes, environmental information processing, genetic information processing, metabolism, and organismal systems. Among these, the metabolic pathways category contained the largest number of unigenes, which fell into 12 major subgroups involved in amino acid metabolism, carbohydrate metabolism, lipid metabolism, glycan biosynthesis and metabolism, energy metabolism and so on (Table 4). In total, 485 sequences were classified into 15 carbohydrate metabolisms pathways, including glycolysis/gluconeogenesis, citrate cycle (TCA cycle), galactose metabolism, pyruvate metabolism, amino sugar and nucleotide sugar metabolism. A total of 446 sequences were classified into 16 lipid metabolism pathways, including arachidonic acid metabolism, fatty acid degradation, sphingolipid metabolism, glycerolipid metabolism, and glycerophospholipid metabolism. A total of 424 sequences were classified into 13 amino acid metabolism pathways, including alanine, aspartate and glutamate metabolism, cysteine and methionine metabolism, glycine, serine and threonine metabolism, tryptophan metabolism, tyrosine metabolism, arginine and proline metabolism. A total of 326 sequences were classified into 8 energy metabolism pathways, including oxidative phosphorylation, methane metabolism, nitrogen metabolism, and sulfur metabolism. Under low salinity conditions, the enzymes involved in glycolysis/gluconeogenesis, oxidative phosphorylation, and arginine and proline metabolism were enriched, indicating that shellfish might resist low salinity through energy metabolism. Another pathway of interest included the subgroups signal transduction. In the KEGG analysis, 1501 sequences were classified into 31 signal transduction pathways, including the AMPK signaling pathway, Calcium signaling pathway, Hippo signaling pathway, MAPK signaling pathway, Notch signaling pathway, PI3K-Akt signaling pathway, Rap1 signaling pathway, Ras signaling pathway, cAMP signaling pathway, Phosphatidylinositol signaling system, Wnt signaling pathway, Neuroactive ligand-receptor interaction, and cGMP - PKG signaling pathway. These pathways may play important roles in the sensing and intracellular transduction of stress signals of *R. philippinarum* under low salinity stress (Fig. 3).

**Table 4:** Kyoto Encyclopedia of Genes and Genomes (KEGG) pathway mapping for *R. philippinarum*.

KEGG Pathways	Sub-Pathways	Number of Unigenes
Cellular Processes	Cell growth and death	406
	Cell motility	174
	Cellular commiunity	492
	Transport and catabolism	780
Environmental Information Processing	Membrane transport	90
	Signal transduction	1501
	Signaling molecules and interaction	372
Genetic Information Processing	Folding, sorting and degradation	602
	Replication and repair	178
	Transcription	290
	Translation	848
Metabolism	Amino acid metabolism	424
	Biosynthesis of other secondary metabolites	75
	Carbohydrate metabolism	485
	Energy metabolism	326
	Glycan biosynthesis and metabolism	264
	Lipid metabolism	446
	Metabolism of cofactors and vitamins	238
	Metabolism of other amino acids	191
	Metabolism of terpenoids and polyketides	56
	Nucleotide metabolism	303
	Xenobiotics biodegradation and metabolism	175
Organismal Systems	Circulatory system	260
	Development	241
	Digestive system	431
	Endocrine system	827
	Environmental adaptation	215
	Excretory system	160
	Immune system	519
	Nervous system	522
	Sensory system	211

### Validation of RNA-Seq results with RT-qPCR

**Figure 7:**
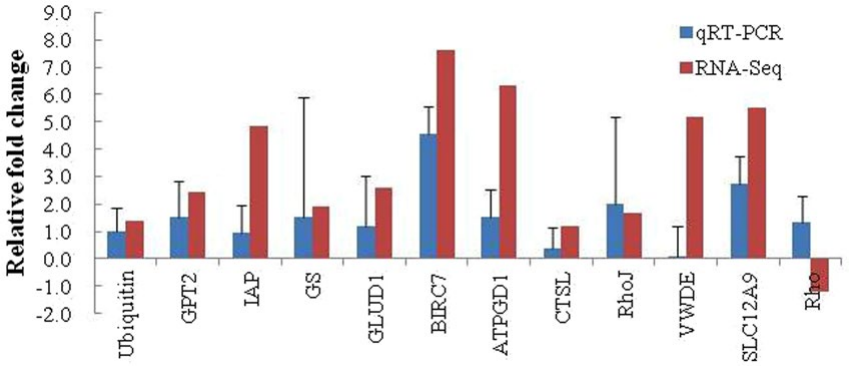
Validation of RNA-Seq results using RT–qPCR. The transcript expression levels of the selected genes were normalized to that of the β-actin gene.

To confirm the differentially expressed genes identified in the RNA-Seq expression analysis, we selected 12 genes from the gene expression heatmap for RT–qPCR validation from those with differing expression patterns among the genes of interest based on their functions. The primers were designed according to the contig sequences (Table 5). A melting-curve analysis revealed a single product for all the tested genes, indicating the high reliability of the transcriptome assembly. The fold changes detected with RT–qPCR were compared with those detected with the RNA-Seq expression analysis (Fig. 7). As shown in Fig. 7, the RT–qPCR results correlated significantly with the RNA-Seq results (*P* < 0.05). In general, the RNA-Seq results were confirmed by the RT–qPCR results, indicating the reliability and accuracy of the RNA-Seq expression analysis.

**Table 5:** Combination of primers used in RT-qPCR assays.

Gene	Gene ID	Oligonucleotide	Sequence
BIRC7	c132162_g1	Forward primer	CATCTTGTTGTGCAGGGTTG
		Reverse primer	TTCCGAGTTAAGAGCGCAAT
GPT2	c144470_g2	Forward primer	ATCTGGGCTGGATGGATGT
		Reverse primer	GTGCTGGACAAAGTGTTGGTT
ATPGD1	c135860_g1	Forward primer	ATGTTCGGTCGACAGTTTCC
		Reverse primer	TTAGATCCGCCGAAATCTTG
Cathepsin L	c154587_g1	Forward primer	TATTTCTGACCGCCCATTTC
		Reverse primer	TCCCATAAGTCCCTGTCGTT
RhoJ	c132087_g1	Forward primer	TGGCTACTCAAACTGACCTGA
		Reverse primer	ACAAATGAATCCGCACCA
IAP	c149735_g1	Forward primer	TTCCAACAGACGACATCCAA
		Reverse primer	GTTTGTACTCGGCCAAGAGG
VWDE	c135387_g1	Forward primer	CCCGATTCTACGCATTTACC
		Reverse primer	GACCTCCAACCAACAGTCGT
SLC12A9	c151354_g1	Forward primer	AACTCTGGCGGTCATTCTTG
		Reverse primer	TTGCTTTCGGCACTTCAAC
GLUS	c139767_g1	Forward primer	GAGTCGGTCAATAGCCTCCT
		Reverse primer	CAAACCAATGCCAGGTGA
GLUD1	c153037_g1	Forward primer	CCCAGCACAGTCAACACAG
		Reverse primer	GTCCACAACGGCACATTTAT
Ubiquitin	c140036_g1	Forward primer	CTGCCTTCTGGACCTGTGAG
		Reverse primer	CAAGTGAACGACGAAACAAATAGTC
ARHGEF12	c151340_g2	Forward primer	TAGCAACAGTCCGTGCGTTA
		Reverse primer	GAGTGCCATCATTGGGTGA

## Discussion

High throughput sequencing of RNA transcriptomes were conducted in *R. philippinarum* to identify genes involved in osmotic stress response. Follow-up work will be based on the results gained here as a foundation for more-targeted studies. No previous study has examined the transcriptional response to low salinity stress in the Manila clam. Future studies will use the target genes identified here to examine individuals, strains, and families. To validate the RNA-Seq results, the expression of 12 low-salinity-responsive genes that are involved in the processes of stress response, energy metabolism, substance metabolism, and immune response, was measured with RT–qPCR. These genes may reflect different aspects of the physiological adaptation mechanism to low salinity environments in *R. philippinarum*.

In this study, we identified a series of genes related to osmolality tolerance and low salinity stress. These data showed that the enriched genes related to signal transduction, metabolic processes, responses to stimuli, oxidation-reduction process, and so on, implicating these processes in the regulation of the response to low salinity stress in gills of *R. philippinarum*. Molecular function related to the carbohydrate binding, chitin binding, calcium ion binding were also significantly represented. The important economic traits of resistance and immunity have been the focus of much work on economically valuable marine shellfish^27–30^. The unigene and annotation information from the Nr, Swiss-Prot, GO, and KEGG databases are valuable genomic resources for *R. philippinarum* and will allow further study of the molecular basis of its low salinity resistance and the energetic system.

Osmotic stress tolerance is important for the adaptation and survival of the Manila clam under low salinity stress. Organisms exposed to low salinity experience more oxidative stress than animals living at optimum salinity^12^. The expression of oxidoreductase proteins was indicated insofar as glutamate dehydrogenase 1 (GLUD1), were significantly overexpressed, consistent with the transcriptomic results presented here. In this study, the gene encoding glutamine synthetase (GLUS) and GLUD1 was significantly upregulated under low salinity stress according to the RT–qPCR analysis (Fig. 7), suggesting that the osmolality tolerance of *R. philippinarum* is probably influenced by the nitrogen and glutamate metabolism and the energy homeostasis. GLUD1 is a mitochondrial matrix enzyme, with a key role in the nitrogen and glutamate metabolism and the energy homeostasis, while the GLUS gene plays an essential role in the metabolism of nitrogen by catalyzing the condensation of glutamate and ammonia to form glutamine^31^.

During cell proliferation and tissue repair, materials for cell growth must be provided. In aquatic organisms, adenosine-5′-triphosphate (ATP), the major energy source for cells in the body, is predominantly supplied by a series of metabolic pathways, including glycolysis, the citric acid cycle, and the electron transport chain^32^. Among these, glycolysis occurs (with variations) in nearly all organisms, both aerobic and anaerobic. The wide occurrence of glycolysis indicates that it is one of the most important energy metabolic pathways^31^. It provides not only high-energy compounds like ATP, but also pyruvate, which can be used in the citric acid cycle to generate more ATP, NADH, and FADH_2_^33–35^. In this study, ATP-grasp domain-containing protein 1 (ATPGD1) was upregulated in response to low salinity stress. It has been reported that ATPGD1 is involved in metabolic process and catalyzes the degradation of beta-alanine in KEGG to maintain osmotic equilibrium under hypo-osmotic stress in the oysters^36^. Its expression was found to increase significantly in the *C. gigas* on the 7th day after hypo-osmotic stress, and reached the highest level under the condition with salinity of 10^36^.

Many studies have revealed that intracellular free amino acids (FAAs) predominantly contribute to intracellular osmolality and to cell volume regulation in oysters^37^. The functions of FAAs as osmolytes have been reported by previous studies^38^. In this study, the FAAs metabolic pathways including glycine, alanine, aspartate, glutamate, beta-alanine, arginine, proline, and taurine metabolic pathways were found in the KEGG enrichment analysis, and especially key enzyme genes involved in these pathways, such as ATPGD, CSAD, and P5CS were activated, altering the osmotic status in the bivalves and allowing them to adapt to osmotic stress conditions^39^. These pathways were observed to be expanded in intertidal animals compared with stenohaline terrestrial animals, providing one of the most important explanations for shellfish adaptation to the intertidal zone^8^. Previous studies demonstrated that stress-induced immune changes have been found in many marine invertebrates, including oyster^40, 41^ and mussel^42, 43^. The innate immunity is the only immunological defense mechanism in invertebrate metazoan^44^. In addition, phagocytosis by immune cells is the predominant mechanism of marine invertebrates defense^45^, which is the same to *R. philippinarum*. Up-regulated genes are all related to focal adhesion, NF-kappa B signaling pathway, apoptosis, TNF signaling pathway, and amino acid synthesis and metabolism, so we inferred that these pathways probably play significant roles in the antioxidant response and immune response associated with the low salinity stress response. This study is expect to provides useful genetic resources for further research on marine bivalves osmoregulation and their stress-induced responses to environmental changes.

In summary, we have reported a comprehensive transcript dataset for the manila clam *R. philippinarum*. The transcripts identified and annotated provide valuable genomic resources that extend our understanding of the unique biological characteristics of this economically important species, such as the low salinity tolerance and antioxidant processes of this species. This study has shown that signaling, membrane, transporter activity, receptor activity, macromolecular complex, cellular component organization or biogenesis, and molecular transducer activity are the most highly enriched pathways under hypo-osmotic stress, inferred from the genes differentially expressed at low salinity. To our knowledge, this work is the first to systematically identify the genes that respond to low salinity in *R. philippinarum*, which enriches the gene expression profile data of the species and will allow environmental, molecular, and physiological studies of bivalves to be performed.

## Materials and Methods

### Clam materials and RNA extraction

Two color strains of *R. philippinarum* (zebra and orange) were collected from the Zhangzidao aquaculture farm in Dalian, Liaoning Province, China. The clams with a similar mean shell length averaging at 19.03 ± 0.93 mm, and 20.90 ± 1.34 mm, respectively, were used in this study. After being transported from the field to the laboratory, the clams were cleaned to remove any fouling and were acclimated in aerated 100 L plastic tanks, containing water at 15 °C with salinity of 30 ppt. Other water quality were measured during the experiment (pH: 8.2 ± 0.1, dissolved oxygen: 8.0 ± 0.2 mg/L). The water was exchanged once per day to discharge waste products from marine invertebrates. All the clams were acclimated in lab for two weeks and fed with Spirulina powder daily. The Manila clam is not an endangered or protected species, so no specific permits were required for the study. Forty clams were divided into 2 groups (10 orange and 10 zebra clams for each group). One group was kept in normal salinity water (salt 30 ppt) and used as control (SRp1, SRp2). The other group (FRp1, FRp2) was subjected to salinity change: the salinity decreased for 6 days, 5 ppt per day. After 6 days gradual hypo-osmotic stress, clams were reared in low salinity water (salt 5 ppt) for one day. The water quality did not change during the experiment (Temperature: 15 °C, pH: 8.2 ± 0.1, dissolved oxygen: 8.0 ± 0.2 mg/L). The vitality and motility of the clams suffered no adverse affects when the salinity changed. The gills from orange and zebra clams were collected, respectively, and then immediately frozen in liquid nitrogen and stored at −80 °C until they were processed for RNA extraction.

The total RNA was extracted from 30 mg tissue samples from 1 individual using RNAprep pure Tissue Kit (TianGene, Beijing, China), according to the manufacturer’s protocol. RNA purity was determined with a NanoPhotometer® spectrophotometer (Implen, CA, USA). The RNA concentration was measured with the Qubit® RNA Assay Kit in a Qubit® 2.0 Fluorometer (Life Technologies, CA, USA). RNA integrity was assessed with the RNA Nano 6000 Assay Kit of the Agilent Bioanalyzer 2100 system (Agilent Technologies, CA, USA).

### Library preparation for Transcriptome sequencing

To assess the transcriptomes of the clams and obtain a quantitative and qualitative gene expression database for low salinity stress, four *R. philippinarum* cDNA libraries representing the control group (SRp1, SRp2) and the treated group (FRp1, FRp2) were constructed. The libraries were subjected to Illumina paired-end sequencing, identifying representative transcripts for a wide range of biological processes. A total amount of 3 μg of RNA per sample was used as the input material for RNA sample preparation. The sequencing libraries were generated using the NEBNext® Ultra™ RNA Library Prep Kit for Illumina® (NEB, USA), according to the manufacturer’s recommendations, and index codes were added to attribute the sequences to each sample. To preferentially select cDNA fragments of 150–200 bp, the library fragments were purified with the AMPure XP system (Beckman Coulter, Beverly, USA). The size-selected adaptor-ligated cDNA was incubated with 3 μl of USER Enzyme (NEB, USA) at 37 °C for 15 min, and then at 95 °C for 5 min before PCR. The PCR was performed with Phusion High-Fidelity DNA Polymerase, universal PCR primers, and Index (X) Primer. The PCR products were purified (AMPure XP system) and the quality of the library was assessed with the Agilent Bioanalyzer 2100 system.

### Assembly and annotation

The raw sequences were deposited in the National Center for Biotechnology Information (NCBI) Short Read Archive (SRA) database (http://www.ncbi.nlm.nih.gov/Traces/sra/). After the adapter sequences were removed, and the ambiguous ‘N’ (unknown nucleotide)-containing sequences (N>10%) and low-quality sequences (quality score < 5) were excluded, the remaining clean reads were assembled with the Trinity software, as described for de novo transcriptome assembly without a reference genome. The nonredundant sequences were checked against public databases for homology annotation, including the NCBI nonredundant protein (Nr) and nonredundant nucleotide (Nt) databases (http://www.ncbi.nlm.nih.gov/), Swiss-Prot (http://www.ebi.ac.uk/uniprot/), Gene Ontology (GO) (http://www.geneontology.org/), Clusters of Orthologous Groups (COG) (http://www.ncbi.nlm.nih.gov/COG/), and Kyoto Encyclopedia of Genes and Genomes (KEGG) (http://www.genome.jp/kegg/). If the results for the different databases were conflicting, a priority order of alignments (Nr>Nt>KEGG>Swiss-Prot>GO>COG databases) was followed. The sequences were compared with the Nr, Nt, and Swiss-Prot databases using the BLASTX algorithm, with an E-value cut-off of 10^−10^. GO terms at the second level were used for the GO annotation. The COG and KEGG classifications were performedwith BLASTX, with an E-value cutoff of 10^−5^. Benchmarking Universal Single-Copy Orthologs (BUSCO) was used for completeness score of the transcriptome assembly^46^.

### Differential expression analysis

Before the differential gene expression analysis, the read counts were adjusted for each sequenced library using the edgeR program package with one scaling normalization factor^47^. To determine the differences in the low salinity tolerant and control *R. philippinarum* transcriptomes more accurately, we used DEGseq^48^ to screen the differentially expressed genes with a relation model that chose SRp1 and SRp2 as the control group and compared it with the FRp1 and FRp2 transcriptome library. *P* values < 0.05 and |log2(fold change)|>1were set as the threshold criteria for significantly different expression.

### Real-time RT-PCR confirmation of Illumina sequencing data

To validate our Illumina sequencing data, 12 differentially expressed genes were selected for quantitative RT–PCR (RT–qPCR) analysis, using the same FRp1, FRp2 and SRp1, SRp2 group RNA samples as were used for transcriptome profiling. The primers were designed with the Primer5 software (Premier Biosoft International). The β-actin gene was used as the internal control for the qPCR analysis. RNA (500 ng) from each sample was measured and treated with RQ1 RNase-Free DNase (Promega) to remove any genomic DNA. The cDNA was synthesized from the treated RNA using a reverse transcriptase reagent kit (PrimeScript™ RT reagent Kit, Takara).

The qPCR was performed with SYBR Premix Ex Taq II (Takara). The reactions were carried out in a total volume of 25 μl containing 2.5 μl of diluted cDNA, 2.5 μl of each primer, and 12.5 μl of SYBR Green PCR Master Mix, with the following cycling profile: 95 °C for 15 min for polymerase activation, followed by 40 cycles at 95 °C for 15 s, at 55 °C for 30 s, and at 70 °C for 30 s. Each sample was processed in triplicate in the Roche LightCycler 480 Real-Time PCR System (Roche). All data were analyzed using the 2^−ΔΔCt^ method^49^.
